# PCR analysis of insertion sequences leads to the generation of artefact amplicons

**DOI:** 10.1099/acmi.0.001191.v3

**Published:** 2026-09-11

**Authors:** Clàudia Morros-Bernaus, Ethan R. Wyrsch, Steven P. Djordjevic, Stineke van Houte, David Sünderhauf

**Affiliations:** 1 Environment and Sustainability Institute, University of Exeter, Biosciences, Penryn, TR10 9FE, UK; 2 Australian Institute for Microbiology and Infection, University of Technology Sydney, Ultimo, NSW 2007, Australia

**Keywords:** antimicrobial resistance, ESBL, Genomics, mobile genetic elements, molecular biology, transposons

## Abstract

Insertion sequences (ISs) are small, self-mobilizing DNA elements widespread across prokaryotic genomes, including chromosomes and plasmids. IS elements frequently co-localize with antimicrobial resistance (AMR) genes and mediate their mobilization, often as part of larger genomic structures that encompass multiple IS elements and antibiotic resistance genes.

In this study, we employed Polymerase Chain Reaction (PCR) to amplify DNA sequences containing two copies of an IS*26* element from two *Escherichia coli* ST131 isolates. While the respective PCRs generated products of the expected size, we also observed multiple amplicons of unexpected sizes, which could be misinterpreted as population heterogeneity attributed to IS mobilization. By extracting, re-amplifying and sequencing individual PCR products, we demonstrate that these amplicons of unexpected sizes were indeed artefact products generated during the PCR reaction, likely mediated by within-PCR recombination of the IS*26* sequences. Furthermore, PCRs with equally oriented primers, each located close to an IS*26* element, also generated artefact amplicons.

This research highlights the limitations of using PCR to assess DNA sequences encoding multiple copies of an IS element and therefore, the presence of these genomic structures or the mobilization of the respective IS elements should not be assessed by diagnostic PCR alone but be corroborated with complementary techniques.

## Data summary

**Fig. 1.**
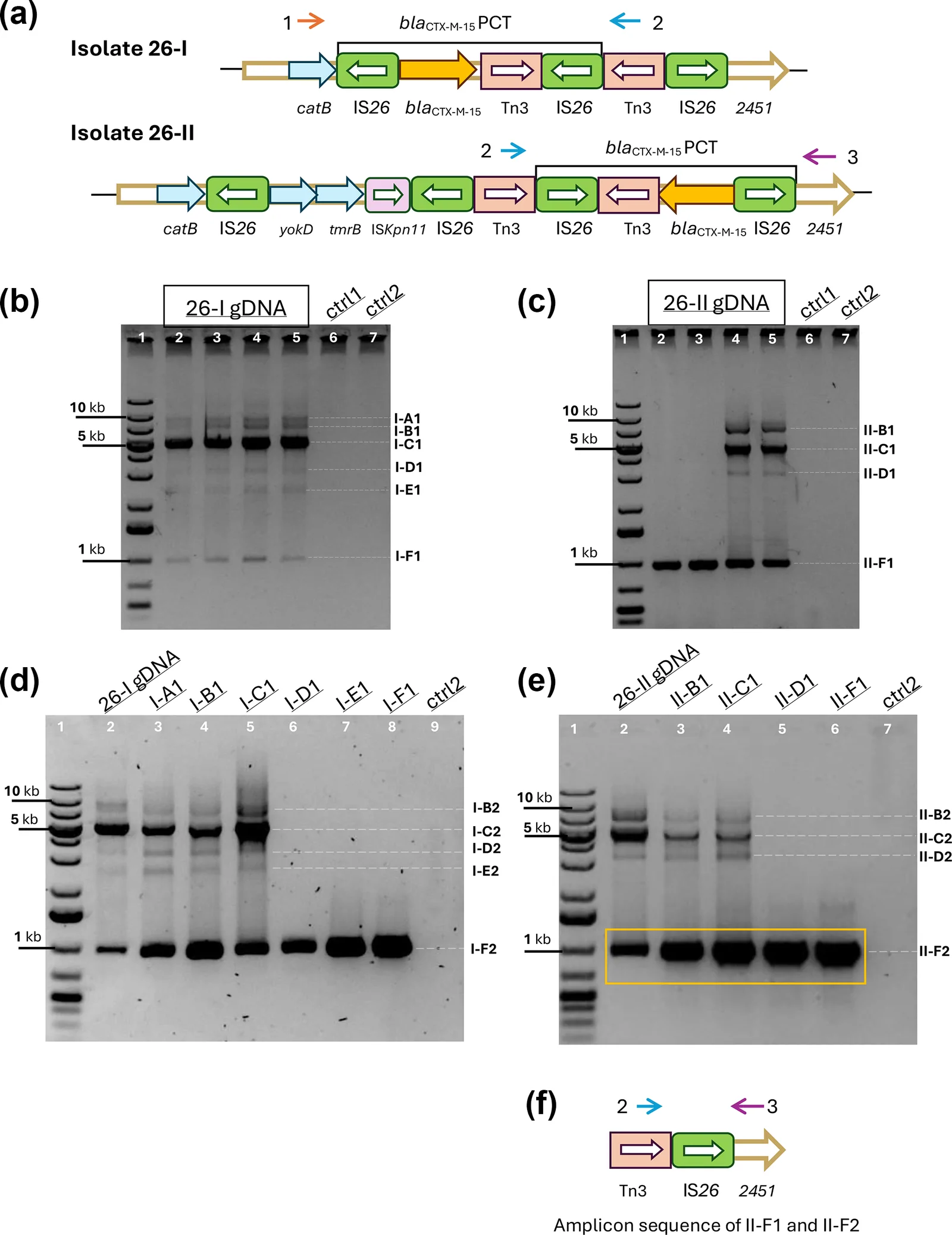
PCR of two pseudo-compound transposons (PCTs) and re-amplification of the multiple PCR products. (**a**) Chromosomal genetic context of the *bla*_CTX-M-15_ PCTs for isolate 26-I (GenBank SAMN49560575 and Supplementary data) and 26-II (GenBank SAMN49560576 and Supplementary data). The pair of primers used to amplify the respective PCTs are shown as arrows and can be found in the PCR section of the methods. (**b–c**) Results from the *bla*_CTX-M-15_ PCT PCR for isolate 26-I (**b**) and 26-II (**c**) when using genomic DNA (gDNA) as template. The amplicons of expected size corresponding to the two respective *bla*_CTX-M-15_ PCTs are 4,950 bp (I-C1) for isolate 26-I and 4,955 bp (II-C1) for isolate 26-II. Ctrl1 is a negative control (*E. coli* DH5α), and ctrl2 is a water control. (**d–e**) Re-amplification PCR using the gel-extracted amplicons recovered from (**b)** for isolate 26-I (**d**) and (**c)** for isolate 26-II (**e**) as DNA templates. The yellow box covers the smallest amplicon, consisting of a single IS*26* element, which were sequenced. (**f**) Visual representation of the sequence corresponding to the II-F1 (GenBank PX317240) and II-F2 amplicons (GenBank PX317240 to PX317244). Genes are shown as arrows and IS elements or transposons as boxes with arrows. *bla*_CTX-M-15_ is shown in yellow and IS*26* in green. *2451 *represents gene *EC958_2451*. Thermo Fisher Scientific GeneRuler 1 kb Plus DNA Ladder was used. Band intensities of representative replicates can be found in Table S1.

**Fig. 2.**
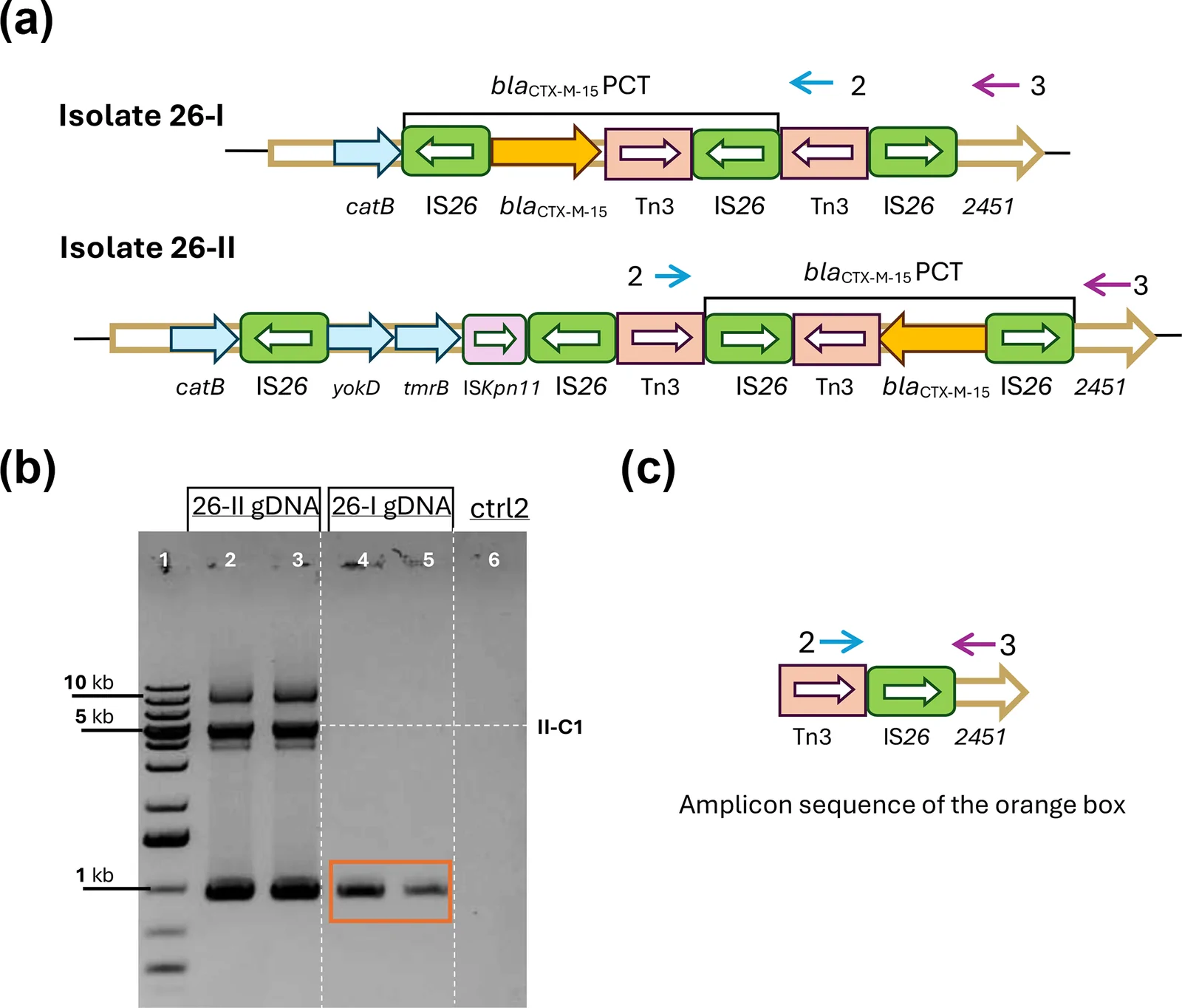
PCR amplification of IS*26*-containing sequences using an equally oriented pair of primers results in the presence of amplicons generated through *in vitro* recombination between the two copies of the IS*26* element. (**a**) IS*26*-containing sequences for isolates 26-I (GenBank SAMN49560575 and Supplementary data) and 26-II (GenBank SAMN49560576 and Supplementary data). Primers 2–3 amplify the *bla*_CTX-M-15_ PCT for isolate 26-II and should not generate a PCR product for isolate 26-I. (**b**) Results from the PCR of isolates 26-II and 26-I. The orange box indicates the amplicons that were sequenced. Ctrl2 is a water control. Band intensities of representative replicates can be found in Table S1. (**c**) Visual representation of the *in vitro* generated PCR product (GenBank PX317246). Genes are shown as arrows and IS elements or transposons as boxes with arrows. *bla*_CTX-M-15_ is shown in yellow, and IS*26* in green. *2451* represents gene *EC958_2451*. The Thermo Fisher Scientific GeneRuler 1 kb Plus DNA Ladder was used. Dotted white lines are used to separate samples with different DNA templates. The PCR was performed using DNA templates 26-II gDNA, 26-I gDNA and 1/10 26-I gDNA; the gel image shown here is a cropped version of the original picture that included additional samples; see Fig. S2.

This research generated sequencing data from Sanger-sequenced PCR amplicons. All the FASTA sequences are deposited in GenBank (PX317240 to PX317246). PX317240 (II-F2 amplicon from 26-II gDNA Fig. 1e), PX317241 (II-F2 amplicon from II-B1 sample Fig. 1e), PX317242 (II-F2 amplicon from II-C1 sample Fig. 1e), PX317243 (II-F2 amplicon from II-D1 sample Fig. 1e), PX317244 (II-F2 amplicon from II-F1 sample Fig. 1e), PX317245 (purple box from 26-II gDNA 1/10 sample Fig S1b, available in the online Supplementary Material) and PX317246 (orange box from 26-I gDNA 1/10 sample Fig. 2b).

## Introduction

 Prokaryotic genomes carry a wide diversity of mobile genetic elements (MGEs), which play an important role in gene mobilization and bacterial evolution. Insertion sequences (ISs) are among the simplest MGEs found widely distributed across prokaryotic genomes [1–3]. These are typically small (0.7–2.5 kb) DNA elements [2, 3]. In fact, more than 4,000 IS elements have been identified and classified into at least 29 different families [2, 3]. A unifying feature of IS elements is that they carry at least one transposase (Tnp, IS-encoded transposase), which mediates their self-mobilization. Transposition of IS elements can contribute to many genomic rearrangements, such as gene mobilization, gene inactivation or modulation of gene expression [2, 4].

IS elements can not only mobilize themselves but also nearby genes, such as antibiotic resistance genes [5]. While this mobilization can be mediated by a single IS element, it often occurs as a composite transposon. This consists of a DNA segment that moves as a single unit and carries the relevant genes flanked by two IS elements, usually of the same type [5]. Composite transposons formed by many IS elements from different families, with the two copies of the IS element oriented either in the same direction (directly-oriented) or opposite directions (inversely-oriented), have been identified [5, 6]. Among IS elements, IS*26* is a clinically relevant and well-known driver of antimicrobial resistance (AMR) mobilization [7] and genome reorganization [8–10]. Upon mobilization, IS*26* preferably inserts next to an existing IS*26* copy, generating composite transposons [11] and composite transposons of directly-oriented IS*26* elements are called pseudo-compound transposons (PCTs) [12].

Polymerase Chain Reaction (PCR) is a widely used method for detecting the presence of specific genes. However, when amplifying DNA templates containing repetitive sequences, artefact PCR products can be generated [13, 14]. PCR amplification of IS-containing regions has been used to track the presence of composite transposons [15–17]. As these regions, by their own nature, contain strongly repetitive sequences, it is important to evaluate whether the homology between the two copies of the same IS element might lead to the generation of such artefact PCR products.

Here, we performed PCR of two chromosomally located IS*26* PCTs of *E. coli* ST131 and observed the emergence of multiple PCR products of unexpected sizes, including an amplicon of a size corresponding to a single IS*26*, which could be interpreted as chromosomal excision of the respective IS*26* PCTs in the ST131 isolates [18–20]. Nevertheless, by extracting and re-amplifying the PCR products, we demonstrate that these amplicons are artefact products generated in the PCR reaction itself. Similarly, we performed PCRs using equally oriented primers located close to IS*26* elements, which also led to the generation of amplicons of unexpected sizes, further indicating within-PCR recombination between IS*26* sequences. This study highlights the limitations of PCR for tracing composite transposons, revealing the need to use complementary techniques to confirm the results or alternative methodologies.

## Results

### PCR amplification of IS*26* pseudo-compound transposons (PCTs) in *E. coli* ST131 isolates leads to multiple amplicons of unexpected sizes

We investigated the presence of *bla*_CTX-M-15_ PCTs (IS*26–bla*_CTX-M-15_–IS*26*) in the *E. coli* ST131 isolates 26-I and 26-II, for which whole-genome sequencing data and a well-characterized *bla*_CTX-M-15_ genetic context are available (BioProject PRJNA1281408 and Supplementary data, [21]). We designed pairs of primers that bind to sequences flanking the PCTs (Fig. 1a), which should for each of the isolates result in amplicons of sizes 4,950 and 4,955 bp, respectively and carried out multiple steps of PCR optimization (Methods, Table 1). After high-fidelity PCR using VeriFi Hot Start polymerase, these amplicons were successfully identified for both isolates (Fig. 1b band I-C1 and Fig. 1c band II-C1); however, even under optimized PCR conditions, PCT amplification was inconsistent, as observed for isolate 26-II (Fig. 1c). For both isolates, the presence of the *bla*_CTX-M-15_ PCT amplicons appeared together with multiple larger and smaller PCR products of similar or lower DNA band intensity compared to the PCT amplicon (Fig. 1b, c and Table S1). In the case of isolate 26-II, the smallest amplicon band (II-F1) was more intense than the desired II-C1 band. Sanger sequencing of this smallest amplicon of isolate 26-II (Fig. 1c*,* band II-F1) revealed the amplification of a single IS*26* element with the appropriate PCT chromosomal genetic context (visual summary in Fig. 1f; sequencing data deposited in GenBank PX317240). This amplicon could indicate an excision of the *bla*_CTX-M-15_ PCT in the *E. coli* ST131 26-II population, since mobilization of IS*26* PCTs leads to a single IS*26* element at the original location [18–20]. Therefore, the presence of such additional PCR products could indicate a heterogeneous bacterial population from which the DNA was extracted and used as a template for PCR, where some individuals may possess alternative PCT forms derived from IS*26* mobilization or PCT loss. Furthermore, some of these products exhibited DNA band intensities comparable to those of the *bla*_CTX-M-15_ PCT amplicons (Table S1). However, analysis of the whole-genome sequencing data for the two isolates, including a detailed assessment of reads across the *bla*_CTX-M-15_ region, revealed no evidence of *bla*_CTX-M-15_ mobilization.

Several authors have previously demonstrated the generation of artefact PCR products when amplifying DNA templates containing repetitive sequences [13, 14, 22]. Therefore, we hypothesized that alternatively these additional PCR products could have arisen from *in vitro* recombination in the PCR reaction, likely facilitated by the presence of two copies of the IS*26* element that have 100% sequence identity.

### DNA re-amplification from gel-extracted amplicons resulted in the same pattern of multiple PCR products

To determine the origin of the multiple amplicons, and particularly of the one containing a single IS*26* element, we gel-extracted all amplicons from the first round of PCRs (Fig. 1b*,* bands I-A1 to I-F1 and Fig. 1c, bands II-B1 to II-F1) and used them individually as DNA templates in a second round of PCRs.

Re-amplification of large amplicons (amplicons A to C) reproduced the same pattern of multiple-sized amplicons (Fig. 1d, e), likely demonstrating that these products were not pre-existent in the wild-type population but emerged *in vitro* during the PCR reaction. In contrast, amplicons smaller than the *bla*_CTX-M-15_ PCT amplicon (amplicons D to F) produced only a single short product.

We Sanger-sequenced the smallest amplicon recovered from each of the different DNA templates for isolate 26-II (Fig. 1e**,** IIF-2, yellow box), which again revealed the presence of a single IS*26* element surrounded by the expected chromosomal context of the PCT (visual summary in Fig. 1f; sequencing data deposited in GenBank PX317240 to PX317244). Sequencing of the other amplicons was not possible due to sub-threshold DNA concentrations, even after re-amplification of purified amplicons, presumably since the PCR reactions favoured the presence of the smallest amplicon. In fact, the single IS*26* element amplicon often emerged at the highest intensity (Table S1). The expected *bla*_CTX-M-15_ PCT amplicon and larger amplicons regenerated all observed amplicon sizes (Fig. 1d, samples A to C and Fig. 1e, samples B to C), including the *bla*_CTX-M-15_ PCT amplicon, indicating the presence of at least two IS*26* elements in the original PCR products. In contrast, amplicons smaller than the *bla*_CTX-M-15_ PCT amplicon (Fig. 1d, samples D to F and Fig. 1e, samples D to F) produced only the simplest single IS*26*-containing amplicon, suggesting the presence of at least one IS*26* element in the original PCR products.

Together, this indicates that the generation of multiple PCR products occurs *in vitro* during the PCR reaction and are therefore artefact products of amplification. All re-amplified amplicons generated at least the simplest amplicon consisting of a single IS*26* element (Fig. 1d, I-F2 and Fig. 1e, II-F2). We observed a size threshold, where only large amplicons – those amplicons likely containing two copies of the IS*26* element – can result in the full spectrum of different-sized PCR products. The emergence of these artefact products likely resulted from an *in vitro* recombination mediated by the homology present between the two copies of the IS*26* element.

### *In vitro* recombination between copies of an IS*26* element generates false-positive amplicons when using pairs of primers with equal orientation

After observing the emergence of multiple amplicons likely resulting from *in vitro* recombination between the two copies of the IS*26* element of the *bla*_CTX-M-15_ PCTs, we hypothesized that *in vitro* recombination could also facilitate the generation of false-positive amplicons. To further study this, we crossed the previously used pairs of primers with the two different isolates, which led to equally oriented pairs of primers for each isolate (Figs 2a and S1a). While equally oriented primers do not usually generate a PCR amplicon, we consistently generated amplicons when performing these equally-oriented-primer PCRs (Figs 2b and S1b). These amplicons were generated at an intensity comparable to the *bla*_CTX-M-15_ PCT amplicon (Table S1). Sanger sequencing revealed the presence of a single IS*26* element surrounded by a chromosomal context that is consistent with *in vitro* recombination between IS*26* sequences (visual summary in Figs 2c and S1c, respectively; Sequencing data deposited in GenBank PX317246 and PX317245). These data are consistent with *in vitro* generation of PCR products through recombination of IS*26* copies between two single-stranded DNA fragments generated by each of the equally oriented primers, followed by further amplification of recombined fragments.

## Discussion

In this study, we present evidence that amplification of a locus containing repetitive sequences can lead to artefact amplicons generated *in vitro* in the PCR reaction. We amplified two *bla*_CTX-M-15_ pseudo-compound transposons (PCTs) carrying two copies of an IS*26* element with 100% sequence identity from two *E. coli* ST131 isolates. In doing so, we identified the expected-size amplicon together with the emergence of multiple larger and smaller PCR products. These unexpected PCR products, particularly the smallest amplicon, arose consistently across PCR conditions (Methods, Table 1). Sanger sequencing of the smallest amplicon revealed the presence of a single IS*26* element, which could be misinterpreted as a chromosomal excision of the *bla*_CTX-M-15_ PCTs in the respective *E. coli* ST131 populations, since it leads to a single IS*26* chromosomal copy [18–20]. However, no evidence of mobilization of the respective *bla*_CTX-M-15_ PCTs was identified from the whole-genome sequencing data. By gel-extracting and re-amplifying the different PCR products we observed the re-emergence of the multiple PCR products, demonstrating that they are not representative of heterogeneity of the *bla*_CTX-M-15_ PCT in the ST131 wild-type populations, but are PCR-mediated artefacts generated *in vitro*. Comparison of the DNA band intensities of the multiple PCR products showed similar amplification efficiencies for the *bla*_CTX-M-15_ PCTs amplicons and the artefact single IS*26* element product, revealing a tendency for preferential generation of the smallest product. We also demonstrated the generation of artefact amplicons when using equally oriented pairs of primers, which likely resulted from *in vitro* recombination between two copies of the IS*26* element located in single-stranded DNA intermediates generated by each respective primer. These artefact products showed DNA bands of comparable intensity to the corresponding *bla*_CTX-M-15_ PCTs amplicons, indicating that they were amplified with similar efficiency. Overall, the high amplification efficiency of artefact PCR products makes them difficult to distinguish from authentic amplification products.

We do not experimentally demonstrate a mechanism of recombination, but our results are reflective of recombination events observed during amplification of transcription activator-like effectors (TALEs) and homologous genes [14] and may therefore reflect a similar recombination model. Under this model, the multiple PCR products would result from incomplete single-stranded IS*26*-containing fragments that act as mega primers or DNA templates, anneal to other IS*26* copies in the subsequent cycles and are amplified. We could not avoid artefact PCR products despite using a VeriFi Hot Start polymerase, which is a highly robust DNA polymerase with increased PCR success in long and challenging templates, according to the manufacturer’s claim. Although we did not investigate strategies to reduce the occurrence of artefact amplicons, previous studies have examined the influence of various buffers, DNA polymerases, DNA binding proteins and DNA template concentrations on the presence of artefact amplicons. Nevertheless, none of the tested conditions fully eliminated the generation of artefact amplicons during amplification [14]. In any case, the use of distantly located primers from the repetitive sequences resulted in a good optimization strategy [14], which should be considered by authors. Besides, the combination of low DNA template concentrations together with a reduced number of PCR cycles has also been reported to reduce the formation of artefact products [23].

We demonstrated the generation of artefact PCR products during the amplification of PCTs from two *E. coli* ST131 isolates. Our results are likely generalizable as key experimental steps are carried out *in vitro* independent of host background, but further studies examining amplification of additional PCT structures and from diverse bacterial isolates are needed to confirm the broader applicability of these findings. The generation of artefact PCR products resulting from the presence of repetitive sequences in the DNA template is not exclusive to ISs in composite transposons and has been previously observed when amplifying homologous genes [13, 14, 22, 24–26], TALEs [14] and CRISPR loci [27]. Importantly, when performing such PCRs, the emergence of an amplicon containing a single copy of the repetitive element is often attributed to *in vivo* recombination between homologous elements. For instance, such amplicons were reported when tracking composite transposons formed by other IS elements from the IS6 family, as well as from other families such as IS1167, IS1191 and IS1193 [17], and when amplifying a DNA element flanked by two copies of *galE* [28]. However, while these studies provide a plausible interpretation, our results indicate that such amplicons could also arise as artefact products generated during the PCR reaction.

Our research demonstrates that PCR of the whole composite transposon is not a reliable technique to study its presence, not only because of the *in vitro* generation of multiple non-specific PCR products but also because it lacks reproducibility, since even under optimal conditions the same sample can lead to different amplification results (Fig. 1c). This is particularly important when assessing composite transposons since the presence of small artefact amplicons resembles the expected product of its excision and could be misinterpreted as evidence of mobilization. Moreover, sequencing of the PCR product does not assist in clarifying this ambiguity, since both wild-type mobilization and *in vitro* PCR recombination result in the exact same sequence. To discern artefact amplicons from a wild-type mobilization of the composite transposons, an additional PCR designed to amplify the circular forms generated upon excision could be performed [16, 29, 30]. However, the primer design for these PCRs needs to be cautiously considered since they are often located near IS elements, and false positive amplicons for the excised circular molecule could be generated in the presence of the chromosomally integrated composite transposon through *in vitro* recombination, similar to the amplicons generated in this study using equally oriented pairs of primers (Figs 2 and S1).

Overall, we encourage authors working with DNA elements containing repetitive sequences to cautiously interpret the results of PCR amplifications. Besides, in the case of composite transposons, it is advisable to perform PCR with one primer located within the composite transposon and another primer located in the flanking chromosomal region, as performed in [31], thereby avoiding the amplification of multiple homologous elements. Alternative *in vivo* approaches such as entrapment vectors [32] could also be employed to distinguish between PCR-mediated artefacts and genuine mobilization of the DNA element.

## Methods

### Bacteria

The two *E. coli* ST131 isolates used were 26-I and 26-II (PRJNA1281408 [21]). Isolate 26-I carries a complex antibiotic-resistant structure formed by four IS*26* elements, whereas isolate 26-II carries a similar complex antibiotic-resistant structure formed by five IS*26* elements. The two isolates carry the same *bla*_CTX-M-15_ PCT but are located in a different chromosomal genetic context. For the two isolates, an assessment of the reads across the *bla*_CTX-M-15_ region was performed using minimap2 [33] and Tablet [34] to study the potential mobility of the composite transposons.

### PCR

For each isolate and pair of primers, DMSO concentrations (0 and 4%), DNA template concentrations and different polymerases were tested (Table 1). From the tested conditions, we selected those that achieved best amplification of the *bla*_CTX-M-15_ PCTs for each isolate. Therefore, PCRs of the respective *bla*_CTX-M-15_ PCTs per isolate were performed using VeriFi Hot Start Polymerase Mix Red (PCR Biosystems) and were carried out according to the manufacturer’s protocols with the following alterations: The mix was used at a final concentration of 0.5× instead of the recommended 1×, since it achieved an optimized amplification of the expected-size amplicon. For isolate 26-I, the first-round amplification of the *bla*_CTX-M-15_ PCT (4,950 bp) and the second-round amplification of all the multiple amplicons recovered from the first PCR were performed using primers 1–2 and 4% DMSO. For isolate 26-II, the first-round amplification of the *bla*_CTX-M-15_ PCT (4,955 bp) and the second-round amplification of all the multiple amplicons recovered from the first PCR were performed using primers 2–3 and 0% DMSO. The PCR cycle conditions were the same for the two isolates: 1× (95 °C 2 min)–30× (95 °C 15 s, 72 °C 2:50 min)–1× (72 °C 7 min). The DNA template used in the first-round PCRs was genomic DNA from isolates 26-I or 26-II, while in the second-round PCRs it was either genomic DNA or gel-extracted PCR products, as specified in the figures. The extraction of the genomic DNA was performed from lysogeny broth (LB) overnight cultures of the respective isolates, which were grown at 37 °C, 180 r.p.m., using QIAamp DNA Mini Kit (Qiagen) following the manufacturer’s instructions. PCRs using equally oriented pairs of primers were performed using the same PCR conditions for each pair of primers and the extracted 26-I and 26-II gDNA. One microlitre of either non-diluted or 1/10 diluted gDNA was used as indicated. To confirm consistency of the results, all PCRs were performed in duplicate. For each replicate, the same aliquot of gDNA for isolate 26-I or 26-II was used, respectively. In the second-round PCRs, which use the amplicons from the first-round PCR as DNA templates, the amplicons from each respective first-round PCR replicate were individually purified and re-amplified.

**Table 1.** PCR polymerases and amplification conditions evaluated to optimize amplification of the respective *bla*_CTX-M-15_ PCTs from isolates 26-I and 26-II. The polymerases tested included Taq Mix Red, VeriFi Mix Red and HotStart VeriFi Mix Red polymerases (PCR Biosystems) and the Phusion High-Fidelity PCR Kit (Thermo Fisher Scientific). Extension times were selected according to the manufacturer’s recommendations. All PCR reactions were performed in a final volume of 10 µl. Glycerol stock DNA template was prepared by scraping material from 20% glycerol frozen stocks of the respective bacterial isolates into 10 µl of sterile H_2_O, followed by incubation at 95 °C for 15 min and centrifugation at 3,500 r.p.m. for 5 min. One microlitre of the supernatant was used as the PCR template. For reactions using gDNA, 1 µl of the purified gDNA was used as the template. The DNA bands column displays the bands observed on the gel corresponding to those characterized in Fig. 1 (shown in parentheses); the desired 4.9 kb band corresponding to the respective *bla*_CTX-M-15_ PCTs is shown in bold

PCR of the bla*_CTX-M-15_* PCT (4,950 bp) from isolate 26-I using primers 1–2					
**Enzyme (concentration)**	**Conditions** **(second step, ×30 cycles)**	**DMSO (%)**	DNA template	**Primer concentration (nM)**	DNA bands
Taq Mix Red (1×)	95 °C 2′–(95 °C 15″–62/64/66 °C 15″–72 °C 2′)–72 °C 7′ Same outcome across annealing temperatures (T_a_s)	0	Glycerol stock and gDNA	400	1 kb (I-F1)
VeriFi Mix Red (1×)	95 °C 2′–(95 °C 15″–68/70/72 °C 15″–72 °C 2:30′)–72 °C 7′ Same outcome across T_a_s	0	Glycerol stock	400	1 kb (I-F1), faint **4.9** kb (I-C1)*
VeriFi Mix Red (1×)	95 °C 2′–(95 °C 15″ – 68/70/72 °C 30′–72 °C 2:30′)–72 °C 7′. Same outcome across T_a_s	0	gDNA	400	1 kb (I-F1), **4.9** kb (I-C1), artefact bands around PCT band
VeriFi Hot Start Mix Red (1×)	95 °C 2′–(95 °C 15″–70/72 °C 15″–72 °C 2:30′)–72 °C 7′. Same outcome across T_a_s	0 and 4	Glycerol stock	400	1 kb (I-F1)
VeriFi Hot Start Mix Red (1×)	95 °C 2′–(95 °C 15″–75 °C 15″ –75 °C 2:30′)–72 °C 7′	0 and 4	Glycerol stock	400	1 kb (I-F1)
VeriFi Hot Start Mix Red (0.15×)	95 °C 2′–(95 °C 15″–72 °C 2:50′)–72 °C 7′	0	Glycerol stock	400, 800	No bands
VeriFi Hot Start Mix Red (0.25×)	95 °C 2′–(95 °C 15″–72 °C 2:50′)–72 °C 7′	0	Glycerol stock	400, 800	No bands
VeriFi Hot Start Mix Red (0.5×)	95 °C 2′–(95 °C 15″–72 °C 2:50′)–72 °C 7′	0	Glycerol stock	400, 800	1 kb (I-F1), very faint **4.9** kb (I-C1)
VeriFi Hot Start Mix Red (0.5×)	95 °C 2′–(95 °C 15″–72 °C 2:50′)–72 °C 7′	4	Glycerol stock	400	1 kb (I-F1), faint **4.9** kb (I-C1), artefact bands around PCT band
VeriFi Hot Start Mix Red (0.5×)	95 °C 2′–(95 °C 15″–72 °C 2:50′)–72 °C 7′ Final conditions	4	gDNA	400	1 kb (I-F1), **4.9** kb (I-C1), artefact bands around PCT band (I-A1, I-BI, I-DI and I-EI)
**PCR of the bla*_CTX-M-15_* PCT (4,955 bp) from isolate 26-II using primers 2–3**					
**Enzyme (concentration)**	**Conditions** **(second step, ×30 cycles)**	**DMSO (%)**	**DNA template**	**Primer concentration (nM)**	**DNA bands**
Taq Mix Red (1×)	95 °C 1′–(95 °C 15″–63 °C 15″−72 °C 1:50′) 72 °C 7′	0 and 4	Glycerol stock	400	1 kb (II-F1)
Phusion High-Fidelity (1×)	98 °C 30″–(98 °C 10″–72 °C 2:10′)–72 °C 7′	0 and 4	Glycerol stock	500	No bands
VeriFi Hot Start Mix Red (0.15×)	95 °C 2′–(95 °C 15″–72 °C 2:50′)–72 °C 7′	0	Glycerol stock	400, 800	No bands
VeriFi Hot Start Mix Red (0.25×)	95 °C 2′–(95 °C 15″–72 °C 2:50′)–72 °C 7′	0	Glycerol stock	400, 800	No bands
VeriFi Hot Start Mix Red (0.5×)	95 °C 2′–(95 °C 15″–72 °C 2:50′)–72 °C 7′	0	Glycerol stock	400, 800	1 kb (II-F1), faint **4.9** kb (II-C1), artefact bands around PCT band
VeriFi Hot Start Mix Red (0.5×)	95 °C 2′–(95 °C 15″–72 °C 2:50′)–72 °C 7′ Final conditions	0	gDNA	400	1 kb (II-F1), **4.9** kb (II-C1), artefact bands around PCT band (II-B1 and II-D1)

*Inconsistent across replicates.

One per cent agarose gels were used to visualize the PCR products, and the amplicons of interest were extracted from the gel and purified using the Spin DNA Gel Extraction Kit (New England Biolabs Monarch) following specifications from the manufacturer. The primer sequences 5′−3′ were: (1) gaggcaatggtcatgcccggaatcaagatc, (2) accatgatgccgactatggcgtgctgaatg and (3) cttgctccttgagtatcgttgcgcaccaga. No potential self-dimer formation was identified during primer analysis when using the Multiple Primer Analyzer tool (Thermo Fisher Scientific). Potential cross-primer dimer formation was identified between primers 1 and 2. To confirm primer specificity, *in silico* analysis of potential off-target binding within the 4-IS*26* and 5-IS*26 bla*_CTX-M-15_-containing regions of each isolate was performed using Benchling. The analysis revealed off-target binding only on sequences differing at least by 11 mismatches. GeneRuler 1 kb Plus DNA Ladder (Thermo Fisher Scientific) was used in all gels.

### Gel analysis

GelAnalyzer 26.1 [35] was used to quantify the intensity of the DNA bands. Default lane and band detection parameters were applied, followed by manual refinement of the band assignments. The intensity values reported in Table S1 correspond to the peak intensity value (arbitrary units) calculated by the software. For figures with multiple lanes corresponding to the same DNA template, a representative lane was used for band intensity quantification.

### Sanger sequencing

PCR products were gel-extracted and sequenced using Source Bioscience Sanger DNA Sequencing service.

## Supplementary material

10.1099/acmi.0.001191.v3 Supplementary Material 1.

## Abbreviations

AMR: antimicrobial resistance
gDNA: genomic DNA
IS: insertion sequence
LB: Lysogeny broth
MGE: mobile genetic element
PCR: Polymerase Chain Reaction
PCT: pseudo-compound transposon
TALE: transcription activator-like effector
Tnp: transposase
