# Supplementary material for: PCR analysis of insertion sequences leads to the generation of artefact amplicons
Source: Access Microbiol. 2026 Sep 11;8(9):001191.v3. doi: 10.1099/acmi.0.001191.v3 (PMC13567838; doi:10.1099/acmi.0.001191.v3)
Supplement: Supplementary file 1 [file acmi-8-01191-s001.pdf]

SUPPLEMENTARY DATA

Supplementary figure 1:

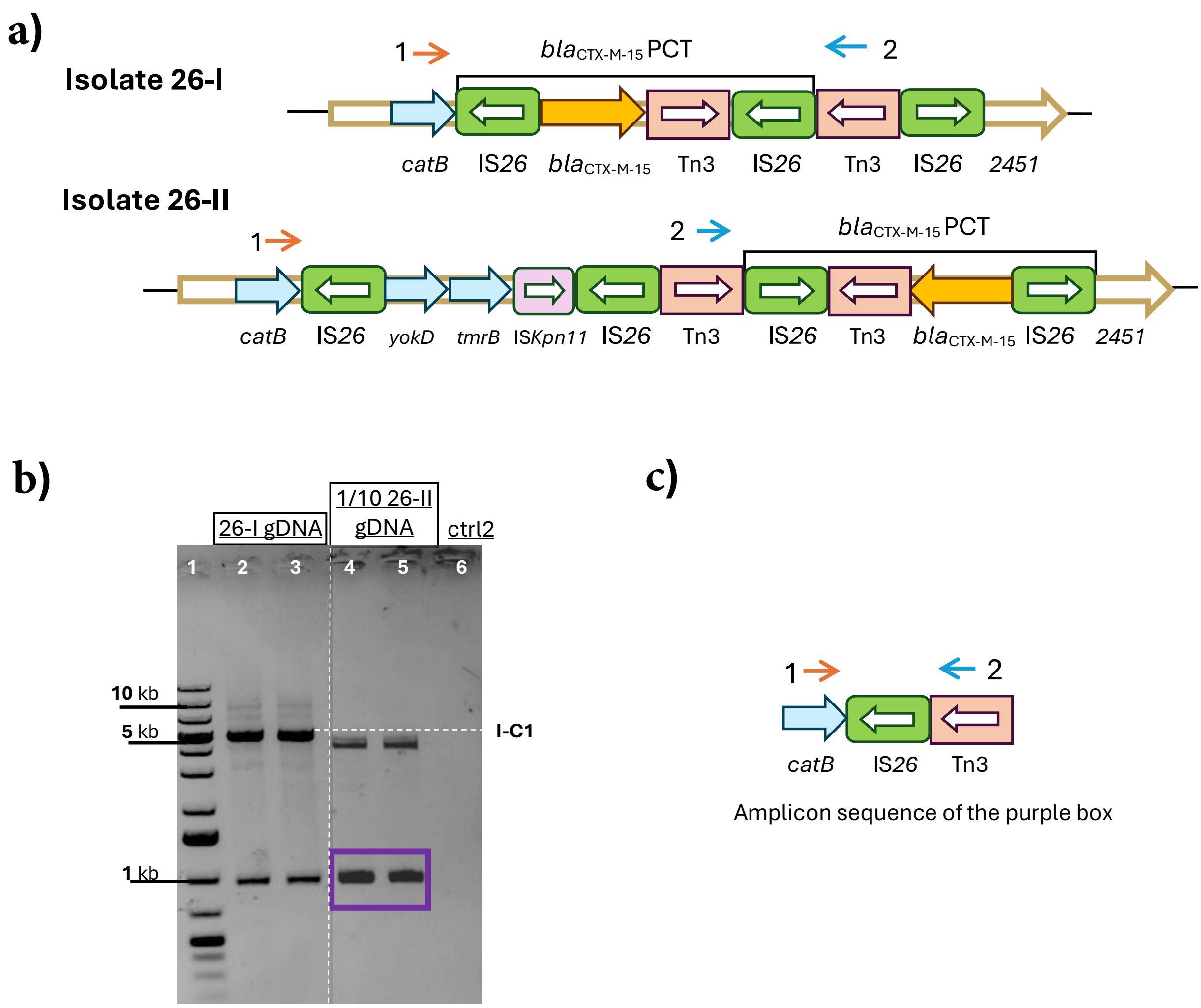

Supplementary figure 1*:** PCR amplification of IS*26*-containing sequences using equally oriented pair of primers results on the presence of amplicons generated through *in vitro* recombination between the two copies of the IS*26* element. **A)** IS*26*-containing sequences for isolates 26-I and 26-II. Primers 1-2 amplify the $bla_{CTX\text{-}M\text{-}15}$ PCT for isolate 26-I and should not give a PCR product for isolate 26-II. **B)** Results from the PCR in isolates 26-I and 26-II. The purple box indicates the amplicons that were sequenced. Ctrl2 is a water control. Band intensities of representative replicates can be found in ***Table S1. **C)** Visual representation of the *in vitro* generated PCR product. Genes are shown as arrows and IS elements or transposons as boxes with arrows. $bla_{CTX\text{-}M\text{-}15}$ is shown in yellow and IS*26* in green. *2451* represents gene *EC958_2451*. GeneRuler 1 kb Plus DNA Ladder was used. Dotted white lines are used to separate samples with different DNA templates. The PCR was performed including DNA templates 26-I gDNA, 26-II gDNA and 1/10 26-II gDNA, the gel image shown here is a cropped version of the original picture which included additional samples, see ***Supplementary figure 3.***

Supplementary figure 2

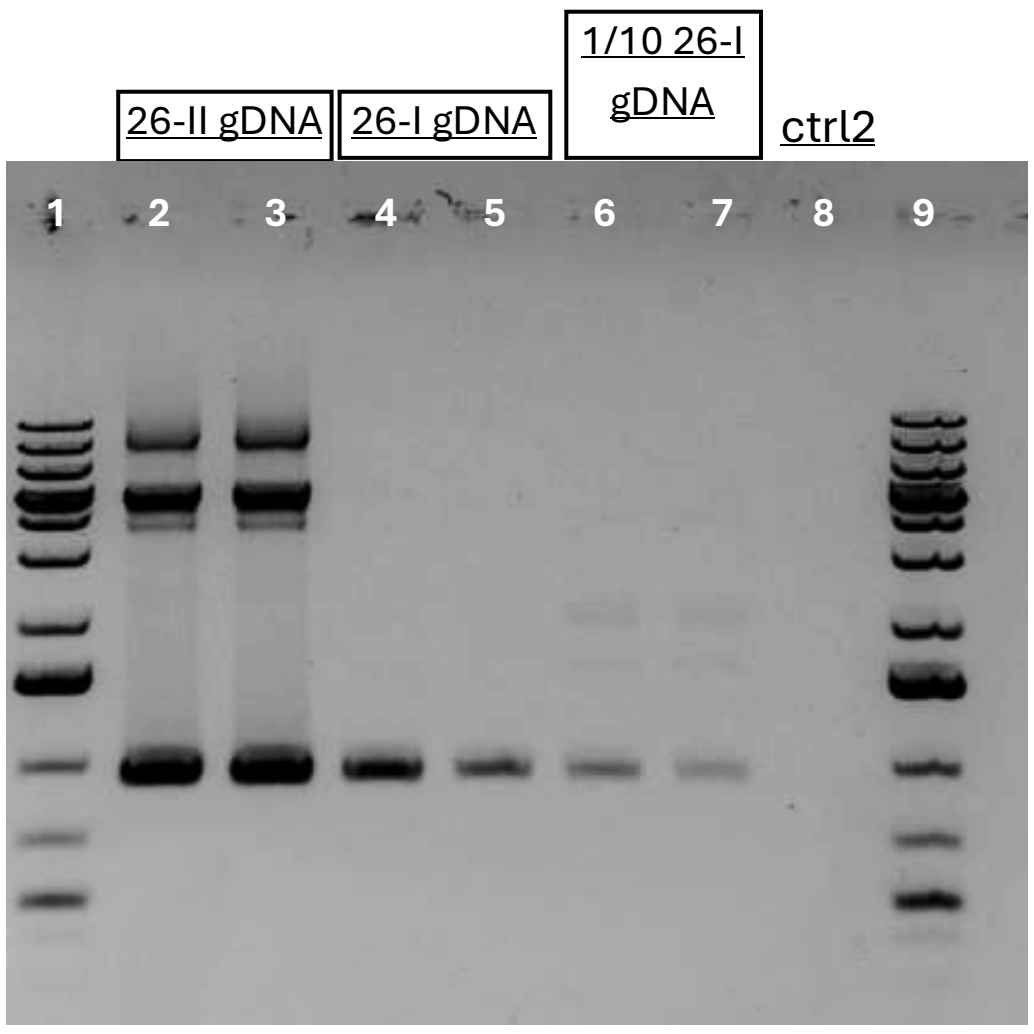

Supplementary figure 2: Full gel from ***Figure 2B*** showing the PCR result when using 26-II gDNA template (lane 2 and 3), 26-I gDNA template (lane 4 and 5) and 1/10 26-I gDNA template (lane 6 and 7). Ctrl 2 is a water control.

Supplementary figure 3

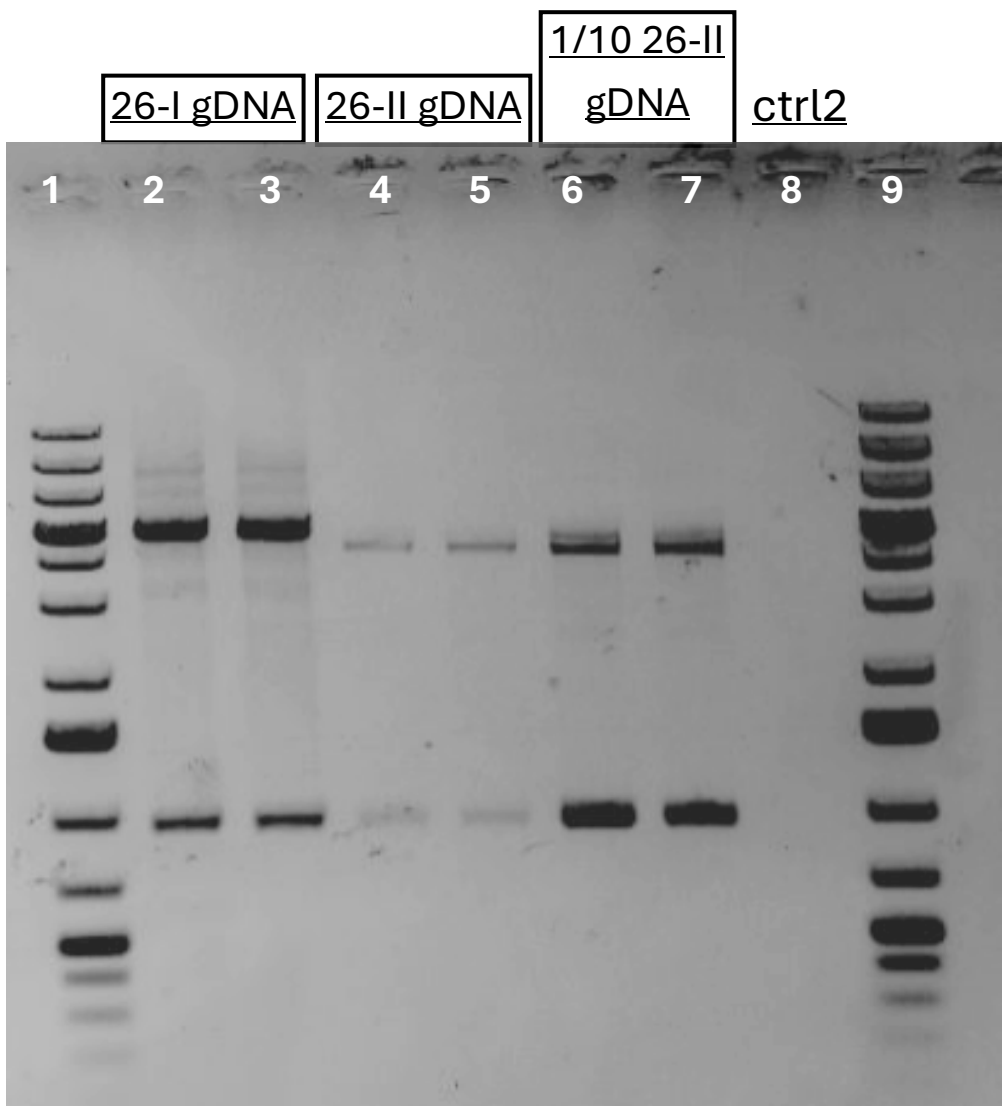

Supplementary figure 3: Full gel from ***Supplementary figure 1*** showing the PCR result when using 26-I gDNA template (lane 2 and 3), 26-II gDNA template (lane 4 and 5) and 1/10 26-II gDNA template (lane 6 and 7). Ctrl 2 is a water control.

Table S1:

**Figure 1B**	**Band**	**Band intensity**
Lane 4	I-A1	17
Lane 4	I-B1	20
Lane 4	I-C1	114
Lane 4	I-D1	5
Lane 4	I-E1	7
Lane 4	I-F1	28
**Figure 1C**	**Band**	**Band intensity**
Lane 2	II-F1	136
Lane 4	II-B1	75
Lane 4	II-C1	119
Lane 4	II-D1	15
Lane 4	II-F1	124
**Figure 1D**	**Band**	**Band intensity**
Lane 5	I-B2	24
Lane 5	I-C2	95
Lane 5	I-D2	16
Lane 5	I-E2	6
Lane 5	I-F2	124
**Figure 1E**	**Band**	**Band intensity**
Lane 4	II-B2	7
Lane 4	II-C2	28
Lane 4	II-D2	12
Lane 4	II-F2	124
**Figure 2B**	**Band**	**Band intensity**
Lane 3	PCT (II-C1)	129
Lane 4	Orange box	145
**Supp. Fig.1B**	**Band**	**Band intensity**
Lane 3	PCT (I-C1)	98
Lane 4	Purple box	95

Table S1: Intensity values of the DNA bands in gels following analysis using GelAnalyzer 26.1. The band intensity column corresponds to the peak intensity value calculated by the software (arbitrary units). For figures with multiple lanes corresponding to the same DNA template, a representative lane was used for band intensity quantification. Band

intensities should be compared within each gel, since the intensities are not normalized across the different gel pictures.

Supplementary sequences

>26-II $bla_{CTX-M-15}$ genetic context visually shown in the figures.

atgaccaactactttgatagccccttcaaaggcaagctgctttctgagcaagtgaagaaccccaatatcaaagttgggcggtacagctattactctggctactatcatgggcactcattc
gatgactgcgcacggtatctgtttccggaccgtgatgacgttgataagttgatcatcggtagtttctgctctatcgggagtggggcttcctttatcatggctggcaatcaggggcatcggtacg
actgggcatcatctttcccgttcttttatatgcaggaagaacctgcattctcaagcgcactcgatgccttccaaaaagcaggtaatactgtcattggcaatgacgtttggatcggctctgag
gcaatggtcatgcccggaatcaagatcgggcacggtgcggtgataggcagccgctcgttggtgacaaaagatgtggggcactgttgcaaagttagcgatgaggcagccttttgtcttattc
aaaggccttacatttcaaaaactctgcttaccaggcgcatttcgcccaggggatcaccataataaaatgctgaggcctggcctttgcgtagtgcacgcatcacctcaatacctttgatgg
tggcgtaagccgtcttcatggatttaaatcccagcgtggcgccgattatccgtttcagtttgccatgatcgcattcaatcacgttgttccggtacttaatctgtcggtgttcaacgtcagacgg
gcaccggccttcgcgtttgagcagagcaagcgcgcgaccataggcgggcgctttatccgtgttgatgaatcgcgggatctgccacttcttcacgttgttgaggattttacccagaaaccgg
tatgcagctttgctgttacgacgggaggagagataaaaatcgacagtgcggccccggctgtcgacggcccggtacagatacgcccagcggccattgaccttcacgtaggtttcatccat
gtgccacgggcaaagatcggaagggttacgccagtaccagcgcagccgtttttccatttcaggcgcataacgctgaacccagcggtaaatcgtggagtgatcgacattcactccgcgtt
cagccagcatctcctgcagctcacggtaactgatgccgtatttgcagtaccagcgtacggcccacagaatgatgtcacgctgaaaatgccggcctttgaatgggttcatgtgcagctcc
atcagcaaaaggggatgataagtttatcaccaccgactatttgcaacagtgcctctcgataggtatagtgttttgcagtttagaggagatatcgcgatgcatacgcggaaggcaataacgg
aggcaattcgaaaattaggagtccaaaccggtgacctgttgatggtgcatgcctcacttaaagcgattggtccggtcgaaggaggagcggagacggtcgttgccgcgttacgctccgcg
gttgggccgactggcactgtgatgggatacgcgtcgtgggaccgatcaccctacgaggagactctgaatggcgctcggttggatgacaaagcccgccgtacctggccgccgttcgatcc
cgcaacggccgggacttaccgtgggttcggcctgctgaatcaatttctggttcaagcccccggcgcgcggcgcagcgcgcaccccgatgcatcgatggtcgcggttggtccgctagctg
aaacgctgacggagcctcacgaactcggtcacgccttgggggaagggtcgcccgtcgagcggttcgtccgccttggcgggaaggccctgctgttgggtgcgccgctaaactccgttac
cgcattgcactacgccgaggcggttgcggatatccccaacaaacgatgggtgacgtatgagatgccgatgcttggaagaaacggtgaagtcgcctggaaaacggcatcagaatacga
ttcaaacggcattctcgattgctttgctatcgaaggaaagccggatgcggtcgaaactatagcaaatgcttacgtgaagctcggtcgccatcgagaaggtgtcgtgggctttgctcagtgct
acctgttcgacgcgcaggacatcgtgacgttcggcgtcacctatcttgagaagcacttcggagccactccgatcgtgccagcacacgaagccgcccagcgctcttgcgagccttccgg
ttagaggccgtcgacaatgataatctggatcaacggacctttcggcgccggaaagacgactctcgctgagcggctgcgcgatcggcgtccgaaatcgctgatctttgaccctgaggaaa
tcgggttcgtggtgaaagaaacggtccccataccagcgagcggagactatcaggatctccccttgtggaggggacttacgatcgctgcggtcagcgagattcgcaggaattactcgcag
gacatcatcatcccaatgacgctcgtgcacccggactatctgactgagatactcgacgggttaaggcagatcgacgatcagctgctgcacatctttctgatgcttaacgaggacctattg
cgtcaccggatctcgaaccagaccatgcatcctgacccgaatcggaatgcggagattcgagagtggcgattagcgaatgtcgcccgatgcttggccgcaagggaacggcttccgtcc
acaacccgtgttctcgatagtggtgcacacaccagcgatgaacttgcagcgatggtgctcgacagactcgatcagcgcacctgatcgccttagagctggctcgggaaatctgcacagc
ggggataagtggagtttctgcctgacttcggcttagatgccgggaacaggagagaccatgacgagacgaccgcgccggaaccatagcccggctttcaaggcgaaggtggcacttgcc
gccatccgaggtgagcagacgctggtggagttgtcccagcagttcgatgtgcacgccaaccagatcaagcaatggaaagaccagctccttgagggggcgacaggtgtgttcggcgatg
aaacgaaagcggagccgtcgggtccgaccatcgatgtcaaaacgctgcacgcgaaaatcggcgaactgacactggagaacgatttttttatccggtgcgctcggcaaggcgggattgc
tgggcggaaagaaatgatcgaccgcgagcacaagctatccgtcgtgcgccaggcgaagcttctcggcttcagccgtggcagcgtctactatctgcctcgtccggtgtctgacggcgatc
tggcccttatgcgccggattgacgaattgcatctcgactacccctttgccggaagtcggatgttgcaagggctcttgagaggagaaggtctggagaccgggcgacacgtcgccacgctg
atgaagaagatgggcatcaaggcgatctaccgccgcccgaacacctcgaaaccagcgccagggcacatacgaaagctggcagtcacccggcccaatcaggtgtgggcaatggac
ctgacctacatccccatggcgcggggatttgtctatctgtgcgccgtcgtggactggttcagccggaaggtcttgtcatggcggttgtcgatcacgatggaagcagccttctgcatcgaggc
ggtggaggaggcacttgcccgtcatggcaggccggaaatcttcaataccgaccagggatcgcagttcacctccatcgacttcaccgccgtgctgaagaggtcacagattgccatctcg
atggatggcaagggtgcgtggcgagacaatgtcttcgtcgagcggctctggcgttcgatcaaatacgaggaagtctacctccatgcctacaagactgtgtccgaggcacgcgctggcat
cgcccgatatctgaacttctacaacaccagacgcccacattggcactgttgcaaagttagcgatgaggcagccttttgtcttattcaaaggccttacatttcaaaaactctgcttaccagg
cgcatttcgcccaggggatcaccataataaaatgctgaggcctggcctttgcgtagtgcacgcatcacctcaatacctttgatggtggcgtaagccgtcttcatggatttaaatcccagcg
tggcgccgattatccgtttcagtttgccatgatcgcattcaatcacgttgttccggtacttaatctgtcggtgttcaacgtcagacgggcaccggccttcgcgtttgagcagagcaagcgcg
cgaccataggcgggcgctttatccgtgttgatgaatcgcgggatctgccacttcttcacgttgttgaggattttacccagaaaccggtatgcagctttgctgttacgacgggaggagagata
aaaatcgacagtgcggccccggctgtcgacggcccggtacagatacgcccagcggccattgaccttcacgtaggtttcatccatgtgccacgggcaaagatcggaagggttacgcca
gtaccagcgcagccgtttttccatttcaggcgcataacgctgaacccagcggtaaatcgtggagtgatcgacattcactccgcgttcagccagcatctcctgcagctcacggtaactgat
gccgtatttgcagtaccagcgtacggcccacagaatgatgtcacgctgaaaatgccggcctttgaatgggttcatgtgcagctccatcagcaaaaggggatgataagtttatcaccacc
gactatttgcaacagtgccataaaatctctgggtcatcagcttgatagtcgttacagacaggttgctgcacgtctttgcgaaaatgaggctgtcgaactcgatgtttctggcccgaagcccc
ggttgacaatttctcccctcgccagtcttgatgagccggacagtctgaaacgactgagcaaaatgatcagtgatctactccctccggtggatttaacggagttgctgctcgaaattaacgc
ccataccggatttgctgatgagtttttccatgctagtgaagccagtgccagagttgatgatctgcccgtcagcatcagcgccgtgctgatggctgaagcctgcaatatcggtctggaacca
ctgatcagatcaaatgttcctgcactgacccgacaccggctgaactggacaaaagcgaactatctgcgggctgaaactatcaccagcgctaatgccagactggttgattttcaggcaa
cgctgccactggcacagatatggggtggaggagaagtggcatctgcagatggaatgcgctttgttacgccagtcagaacaatcaatgccggaccgaaccgcaaatactttggtaataa
cagagggatcacctggtacaactttgtgtccgatcagtattccggctttcatggcatcgttataccggggacgctgagggactctatctttgtgctggaaggtcttctggaacaggagaccg
ggctgaatccaaccgaaattatgaccgatacagcaggtgccagcgaacttgtctttggccttttctggctgctgggataccagttttctccacgcctggctgatgccggtgcttcggttttct
ggcgaatggaccatgatgccgactatggcgtgctgaatgatattgccagagggcaatcagatccccgaaaaatagggcactgttgcaaatagtcggtggtgataaacttatcatcccctt
ttgctgatggagctgcacatgaacccattcaaaggccggcattttcagcgtgacatcattctgtgggccgtacgctggtactgcaaatacggcatcagttaccgtgagctgcaggagatg
ctggctgaacgcggagtgaatgtcgatcactccacgatttaccgctgggttcagcgttatgcgcctgaaatggaaaaacggctgcgctggtactggcgtaacccttccgatctttgcccgt
ggcacatggatgaaacctacgtgaaggtcaatggccgctgggcgtatctgtaccgggccgtcgacagccggggccgcactgtcgatttttatctctcctcccgtcgtaacagcaaagct
gcataccggtttctgggtaaaatcctcaacaacgtgaagaagtggcagatcccgcgattcatcaacacggataaagcgcccgcctatggtcgcgcgcttgctctgctcaaacgcgaag
gccggtgcccgtctgacgttgaacaccgacagattaagtaccggaacaacgtgattgaatgcgatcatggcaaactgaaacggataatcggcgccacgctgggatttaaatccatgaa
gacggcttacgccaccatcaaaggtattgaggtgatgcgtgcactacgcaaaggccaggcctcagcattttattatggtgatcccctgggcgaaatgcgcctggtaagcagagtttttga
aatgtaaggcctttgaataagacaaaaggctgcctcatcgctaactttgcaacagtgccgattttattgcttcctgcgggtctgtcgggtgccccaaagaacgataaaccttaatccggttt
gcctgccagtcagcaccctgtagtaatcttgcacgaggatctccccaccggttactgccggtaacgtagacatccctccgcctcagactatcctgcagtttactgagaaagcagagcgt
gtatcccctgcgggtgatatgtttttccttgttaatcaccagccgtttccatgaccgactgataatttccgttggtgcgtcgtcaaaaaactgccgccgtgagctgaactcccggctgaggta
gtcacaggcattcagagtggtaaccccggcaggtgcggatgaaaatttaacggtattcagcagatggggcaggaaacgacgaacgcgcccgtactgctccaccatttcttcatgaaaa

ttatcgtctgagggccgggcaatttcacggacaagcgtgatgatttcagccagcttttgccttgggatgtagctgaacacctcagcacgaatcgattcgtccggtgtttcttctttcagcagt
acgaacatgcgctggcgagcgccaatgcagatttatccagatccttcagcgagcggagccgtttttttctgcccaatctttctggcgtcacggatgataacggccagcatggcgtccaga
acgtccaatgcatcatccagcgccagcgtttcccatgcaaggacaaaggcaaccagaaccgccatccttttctgcggtgacatcctggcaatattgaacaccgaagtcataccagca
taacgtgcgagatttttcaggcgcacagccgggagtgtactcaggttttcagcatgcaggccaaaatcgttcagagttttccagcgttcaattgcttcattaaacgccggaccactgatggt
cacagggccctttttcagtgattccagtaaagacaggcggctgcaatcagttggccccagcagcatctccagctgtgaacgctgttcggctgacggtatcagtgccagtttgttccacag
gcgcaacgtcgccttttcccttacctctgaaatcaaccgggtcagcgtagtggctccggggagaataatacgatgttgcataagccaccctgtcgccagatcgaaaagcaggccagga
cgttcgttgcttatccagctccgggtatataaaagacgggtaaggcgaaatgtccagggccaggcaaattcacgatactgatagtgctgacgtatcagcgctgcatgctcacggcgggta
ttttccctctgaccgtattctgcaagaacggtgatatcacgaatcccgagctgtctggcggtaaaatgccggacgccggaaggaatatgacctgggacctacgtgcgcccgcaccgac
accctcacaccttcgagctactgttgccattaaggggtcgtttcgtggtgctgaattttgacgatcggggtaccgtcacccatcgggcgatattgggggaaacctgtacggtgctggagatg
gccgcaggaacctggcatgccgtgctgtcgctggataccggtggcataattttttgaagtaaacacggtggctatcaacccgtggctgccgatgactatgcgcactgggctccagcggaa
ggagaaccaggaaccacggagcttatggcctggtatgcgcaagcgcaggtgggcgacagcacttttgccgtctaaggcgataaacaaaaacggaatgagtttccccattccgtttccg
ctattacaaaccgtcggtgacgattttagccgccgacgctaatacatcgcgacggctttctgccttaggttgaggctgggtgaagtaagtgaccagaatcagcggcgcacgatcttttggc
cagatcaccgcgatatcgttggtggtgccatagccaccgctgccggttttatccccccacaacccaggaagcaggcagtccagcctgaatgctcgctgcaccggtggtattgcctttcat
ccatgtcaccagctgcgcccgttggctgtcgcccaatgctttacccagcgtcagattccgcagagtttgcgccattgcccgaggtgaagtggtatcacgcggatcgcccggaatggcgg
tgtttaacgtcggctcggtacggtcgagacggaacgtttcgtctcccagctgtcgggcgaacgcggtgacgctagccgggccgccaacgtgagcaatcagcttattcatcgccacgttat
cgctgtactgtagcgcggccgcgctaagctcagccagtgacatcgtcccattgacgtgcttttccgcaatcggattatagttaacaaggtcagatttttttgatctcaactcgctgatttaaca
gattcggttcgctttcacttttcttcagcaccgcggccgcggccatcactttactggtgctgcacatcgcaaagcgctcatcagcacgataaagtatttgcgaattatctgctgtgttaatca
atgccacacccagtctgcctcccgactgccgctctaattcggcaagtttttgctgtacgtccgccgtttgcgcatacagcggcacacttcctaacaacagcgtgacggttgccgtcgcca
tcagcgtgaactggcgcagtgatttttttaaccatgggattccttattctggaagatacgaaataacaacaacatgaatagtccctaaattccacgtgtgtttttttattagcttcaaaaatcac
tatttcacgaagaatttagactgcttctcacacattgtaacattatttacaaccacctttcaatcatttttgataaatcattgatttcatctttgctgcaatgatacttaataaactctgcaagtt
atccacagagcaacactcaattttattgatgatattcttattataccagacatttttcatacactcccttgtacggatagttttccgacaacttcatgattacatatcttgcggttttgattattttt
gctgcaagaaatacatacttcaaacgaaaggtctttatttgctgtctgtattctgaagagtccaaggaatcaaacttgaacaacaaaaataggttatatgaaagcatcatcatttgaaaca
cggcttcattcgcccaaaatgactttagcaagagatgacccaccgccatgtcgtatttggcttctttgatatagttttcagggcactgttgcaaatagtcggtggtgataaacttatcatccc
cttttgctgatggagctgcacatgaacccattcaaaggccggcattttcagcgtgacatcattctgtgggccgtacgctggtactgcaaatacggcatcagttaccgtgagctgcaggaga
tgctggctgaacgcggagtgaatgtcgatcactccacgatttaccgctgggttcagcgttatgcgcctgaaatggaaaaacggctgcgctggtactggcgtaacccttccgatctttgccc
gtggcacatggatgaaacctacgtgaaggtcaatggccgctgggcgtatctgtaccgggccgtcgacagccggggccgcactgtcgatttttatctctcctcccgtcgtaacagcaaag
ctgcataccggtttctgggtaaaatcctcaacaacgtgaagaagtggcagatcccgcgattcatcaacacggataaagcgcccgcctatggtcgcgcgcttgctctgctcaaacgcga
aggccggtgcccgtctgacgttgaacaccgacagattaagtaccggaacaacgtgattgaatgcgatcatggcaaactgaaacggataatcggcgccacgctgggatttaaatccatg
aagacggcttacgccaccatcaaaggtattgaggtgatgcgtgcactacgcaaaggccaggcctcagcattttattatggtgatcccctgggcgaaatgcgcctggtaagcagagttttt
gaaatgtaaggcctttgaataagacaaaaggctgcctcatcgctaactttgcaacagtgccgaaatactatccaggacagccagggcaggtatttgatgattattacggcggtaaaatct
ggtgcgcaacgatactcaaggagcaaggtgtcggtgcgctggcccgttttgctccttacgctgctggtgacacatgtggtgaagtgttgatgcatatcaaccacccgcaggcacttacact
acttatccatgcaagcgagcagggaaagcgttgccacgatcgcatgacaaagactttcgttcgctttccacatgcagcgctggcggcattagcagaacttctggcacaaaaagatcaa
aagcgttggcgcatgatgctaatgacaatgcttatctcacaaccaacattagcagagcgagtaatcccctggctctcaacacccgcagttgccgtactgaaatcatgccagcaacaac
tgacacagccctcaaaccatgccagcgctgatatgctgcctgccgtattagtctcaccgccctggctttcgaaaaagaaaaaatctgtaatgccggtattggatttaacgccattaccgc
tggagtcatgctgcacattgaccgagacggcagaaaaagagattcatgcaaggcatcgctggcacgcacatcaaatagacatcggccaaaaagaggatattcagaactacctgaca
cgtctgggtttcaatcgctggaataatggccagtatatgaaagcgtctgatgctgttgtagaactct

>26-I *bla*$_{CTX\text{-}M\text{-}15}$ genetic context visually shown in the figures.

atgaccaactactttgatagcccccttcaaaggcaagctgctttctgagcaagtgaagaaccccaatatcaaagttgggcggtacagctattactctggctactatcatgggcactcattc
gatgactgcgcacggtatctgtttccggaccgtgatgacgttgataagttgatcatcggtagtttctgctctatcgggagtggggcttccttttatcatggctggcaatcaggggcatcggtacg
actgggcatcatctttcccgttcttttatatgcaggaagaacctgcattctcaagcgcactcgatgccttccaaaaaagcaggtaatactgtcattggcaatgacgtttggatcggctctgag
gcaatggtcatgcccggaatcaagatcgggcacggtgcggtgataggcagccgctcgttggtgacaaaagatgtggggcactgttgcaaagttagcgatgaggcagccttttgtcttattc
aaaggccttacatttcaaaaactctgcttaccaggcgcatttcgcccaggggatcaccataataaaatgctgaggcctggcctttgcgtagtgcacgcatcacctcaatacctttgatgg
tggcgtaagccgtcttcatggatttaaatcccagcgtggcgccgattatccgtttcagtttgccatgatcgcattcaatcacgttgttccggtacttaatctgtcggtgttcaacgtcagacgg
gcaccggccttcgcgtttgagcagagcaagcgcgcgaccataggcgggcgctttatccgtgttgatgaatcgcgggatctgccacttcttcacgttgttgaggattttacccagaaaccgg
tatgcagctttgctgttacgacgggaggagagataaaaatcgacagtgcggccccggctgtcgacggcccggtacagatacgcccagcggccattgaccttcacgtaggtttcatccat
gtgccacgggcaaagatcggaagggttacgccagtaccagcgcagccgtttttccatttcaggcgcataacgctgaacccagcggtaaatcgtggagtgatcgacattcactccgcgtt
cagccagcatctcctgcagctcacggtaactgatgccgtatttgcagtaccagcgtacggcccacagaatgatgtcacgctgaaaatgccggcctttgaatgggttcatgtgcagctcc
atcagcaaaaggggatgataagtttatcaccaccgactatttgcaacagtgccctgaaaactatatcaaagaagccaaatacgacatggcggtgggtcatctcttgctaaagtcattttg
ggcgaatgaagccgtgtttcaaatgatgatgctttcatataacctatttttgttgttcaagtttgattccttggactcttcagaatacagacagcaaataaagacctttcgtttgaagtatgtatt
tcttgcagcaaaaataatcaaaaccgcaagatatgtaatcatgaagttgtcggaaaactatccgtacaagggagtgtatgaaaaatgtctggtataataagaatatcatcaataaaattg
agtgttgctctgtggataacttgcagagtttattaagtatcattgcagcaaagatgaaatcaatgatttatcaaaaatgattgaaaggtggttgtaaataatgttacaatgtgtgagaagcagt
ctaaattcttcgtgaaatagtgatttttgaagctaataaaaaacacacgtggaatttagggactattcatgttgttgttatttcgtatcttccagaataaggaatcccatggttaaaaaatcact
gcgccagttcacgctgatggcgacggcaaccgtcacgctgttgttaggaagtgtgccgctgtatgcgcaaacggcggacgtacagcaaaaacttgccgaattagagcggcagtcggga
ggcagactgggtgtggcattgattaacacagcagataattcgcaaatactttatcgtgctgatgagcgctttgcgatgtgcagcaccagtaaagtgatggccgcggccgcggtgctgaag
aaaagtgaaagcgaaccgaatctgttaaatcagcgagttgagatcaaaaaatctgaccttgttaactataatccgattgcggaaaagcacgtcaatgggacgatgtcactggctgagct
tagcgcggccgcgctacagtacagcgataacgtggcgatgaataagctgattgctcacgttggcggcccggctagcgtcaccgcgttcgcccgacagctgggagacgaaacgttccgt
ctcgaccgtaccgagccgacgttaaacaccgccattccgggcgatccgcgtgataccacttcacctcgggcaatggcgcaaactctgcggaatctgacgctgggtaaagcattgggc
gacagccaacgggcgcagctggtgacatggatgaaaggcaataccaccggtgcagcgagcattcaggctggactgcctgcttcctgggttgtgggggataaaaccggcagcggtggct
atggcaccaccaacgatatcgcggtgatctggccaaaagatcgtgcgccgctgattctggtcacttacttcacccagcctcaacctaaggcagaaagccgtcgcgatgtattagcgtc

ggcggctaaaatcgtcaccgacggtttgtaatagcggaaacggaatggggaaactcattccgtttttgtttatcgccttagacggcaaaagtgctgtcgcccacctgcgcttgcgcatacc
aggccataagctccgtggttcctggttctccttccgctggagcccagtgcgcatagtcatcggcagccacgggttgatagccaccgtgttttacttcaaaaattatgccaccggtatccag
cgacagcacggcatgccaggttcctgcggccatctccagcaccgtacaggtttcccccaatatcgcccgatgggtgacggtaccccgatcgtcaaaattcagcaccacgaaacgac
cccttaatggcaacagtagctcgaaggtgtgagggtgtcggtgcgggcgcacgtaggtcccaggtcatattccttccggcgtccggcattttaccgccagacagctcgggattcgtgatat
caccgttcttgcagaatacggtcagagggaaaatacccgccgtgagcatgcagcgctgatacgtcagcactatcagtatcgtgaatttgcctggccctggacatttcgccttacccgtct
tttatatacccggagctggataagcaacgaacgtcctggcctgcttttcgatctggcgacagggtggcttatgcaacatcgtattattctccccggagccactacgctgacccggttgattt
cagaggtaagggaaaaggcgacgttgcgcctgtggaacaaactggcactgataccgtcagccgaacagcgttcacagctggagatgctgctggggccaactgattgcagccgcctgt
ctttactggaatcactgaaaaagggccctgtgaccatcagtggtccggcgtttaatgaagcaattgaacgctggaaaactctgaacgattttggcctgcatgctgaaaacctgagtacac
tcccggctgtgcgcctgaaaaatctcgcacgttatgctggtatgacttcggtgttcaatattgccaggatgtcaccgcagaaaaggatggcggttctggttgcctttgtccttgcatgggaaa
cgctggcgctggatgatgcattggacgttctggacgccatgctggccgttatcatccgtgacgccagaaagattgggcagaaaaaacggctccgctcgctgaaggatctggataaatct
gcattggcgctcgccagcgcatgttcgtacctgctgaaagaagaaacaccggacgaatcgattcgtgctgaggtgttcagctacatcccaaggcaaaagctggctgaaatcatcacgc
ttgtccgtgaaattgcccggccctcagacgataattttcatgaagaaatggtggagcagtacgggcgcgttcgtcgtttcctgccccatctgctgaataccgttaaattttcatccgcacct
gccggggttaccactctgaatgcctgtgactacctcagccgggagttcagctcacggcggcagtttttttgacgacgcaccaacggaaattatcagtcggtcatggaaacggctggtgatt
aacaaggaaaaacatatcacccgcaggggatacacgctctgctttctcagtaaactgcaggatagtctgaggcggagggatgtctacgttaccggcagtaaccggtggggagatcctc
gtgcaagattactacagggtgctgactggcaggcaaaccggattaaggtttatcgttctttggggcacccgacagacccgcaggaagcaataaaatcggcactgttgcaaagttagcga
tgaggcagccttttgtcttattcaaaggccttacatttcaaaaactctgcttaccaggcgcatttcgcccaggggatcaccataataaaatgctgaggcctggcctttgcgtagtgcacgc
atcacctcaatacctttgatggtggcgtaagccgtcttcatggatttaaatcccagcgtggcgccgattatccgtttcagtttgccatgatcgcattcaatcacgttgttccggtacttaatct
gtcggtgttcaacgtcagacgggcaccggccttcgcgtttgagcagagcaagcgcgcgaccataggcgggcgctttatccgtgttgatgaatcgcgggatctgccacttcttcacgttgtt
gaggattttacccagaaaccggtatgcagctttgctgttacgacgggaggagagataaaaatcgacagtgcggccccggctgtcgacggcccggtacagatacgcccagcggccattg
accttcacgtaggtttcatccatgtgccacgggcaaagatcggaagggttacgccagtaccagcgcagccgtttttccatttcaggcgcataacgctgaacccagcggtaaatcgtgga
gtgatcgacattcactccgcgttcagccagcatctcctgcagctcacggtaactgatgccgtatttgcagtaccagcgtacggcccacagaatgatgtcacgctgaaaatgccggccttt
gaatgggttcatgtgcagctccatcagcaaaaggggatgataagtttatcaccaccgactatttgcaacagtgccctatttttcggggatctgattgccctctggcaatatcattcagcacg
ccatagtcggcatcatggtccattcgccagaaaaccgaagcaccggcatcagccaggcgtggagaaaactggtatcccagcagccagaaaaggccaaagacaagttcgctggcac
ctgctgtatcggtcataatttcggttggattcagcccggtctcctgttccagaagaccttccagcacaaagatagagtccctcagcgtccccggtataacgatgccatgaaagccggaat
actgatcggacacaaagttgtaccaggtgatccctctgttattaccaaagtatttgcggttcggtccggcattgattgttctgactggcgtaacaaagcgcattccatctgcagatgccactt
ctcctccaccccatatctgtgccagtggcagcgttgcctgaaaatcaaccagtctggcattagcgctggtgatagtttcagcccgcagatagttcgcttttgtccagttcagccggtgtcgg
gtcagtgcaggaacatttgatctgatcagtggttccagaccgatattgcaggcttcagccatcagcacggcgctgatgctgacgggcagatcatcaactctggcactggcttcactagca
tggaaaaactcatcagcaaatccggtatgggcgttaatttcgagcagcaactccgttaaatccaccggagggagtagatcactgatcattttgctcagtcgtttcagactgtccggctcat
caagactggcgaggggagaaattgtcaaccggggcttcgggccagaaacatcgagttcgacagcctcattttcgcaaagacgtgcagcaacctgtctgtaacgactatcaagctgatg
acccagagattttatggcactgttgcaaatagtcggtggtgataaacttatcatccccttttgctgatggagctgcacatgaacccattcaaaggccggcattttcagcgtgacatcattctg
tgggccgtacgctggtactgcaaatacggcatcagttaccgtgagctgcaggagatgctggctgaacgcggagtgaatgtcgatcactccacgatttaccgctgggttcagcgttatgcg
cctgaaatggaaaaacggctgcgctggtactggcgtaacccttccgatctttgcccgtggcacatggatgaaacctacgtgaaggtcaatggccgctgggcgtatctgtaccgggccgt
cgacagccggggccgcactgtcgatttttatctctcctcccgtcgtaacagcaaagctgcataccggtttctgggtaaaatcctcaacaacgtgaagaagtggcagatcccgcgattcat
caacacggataaagcgcccgcctatggtcgcgcgcttgctctgctcaaacgcgaaggccggtgcccgtctgacgttgaacaccgacagattaagtaccggaacaacgtgattgaatg
cgatcatggcaaactgaaacggataatcggcgccacgctgggatttaaatccatgaagacggcttacgccaccatcaaaggtattgaggtgatgcgtgcactacgcaaaggccaggc
ctcagcattttattatggtgatcccctgggcgaaatgcgcctggtaagcagagtttttgaaatgtaaggcctttgaataagacaaaaggctgcctcatcgctaactttgcaacagtgccga
aatactatccaggacagccagggcaggtatttgatgattattacggcggtaaaatctggtgcgcaacgatactcaaggagcaaggtgtcggtgcgctggcccgttttgctccttacgctgc
tggtgacacatgtggtgaagtgttgatgcatatcaaccacccgcaggcacttacactacttatccatgcaagcgagcagggaaagcgttgccacgatcgcatgacaaagactttcgttc
gctttccacatgcagcgctggcggcattagcagaacttctggcacaaaaagatcaaaagcgttggcgcatgatgctaatgacaatgcttatctcacaaccaacattagcagagcgagt
aatcccctggctctcaacacccgcagttgccgtactgaaatcatgccagcaacaactgacacagccctcaaaccatgccagcgctgatatgctgcctgccgtattagtctcaccgcc
ctggctttcgaaaaagaaaaaatctgtaatgccggtattggatttaacgccattaccgctggagtcatgctgcacattgaccgagacggcagaaaaagagattcatgcaaggcatcgct
ggcacgcacatcaaatagacatcggccaaaaagaggatattcagaactacctgacacgtctgggtttcaatcgctggaataatggccagtatatgaaagcgtctgatgctgttgta
